# Predicting Lifetime Suicide Attempts in a Community Sample of Adolescents Using Machine Learning Algorithms

**DOI:** 10.1177/10731911231167490

**Published:** 2023-04-24

**Authors:** Kristin Jankowsky, Diana Steger, Ulrich Schroeders

**Affiliations:** 1 University of Kassel, Germany

**Keywords:** suicide prediction, suicide risk screening, adolescents, machine learning

## Abstract

Suicide is a major global health concern and a prominent cause of death in adolescents. Previous research on suicide prediction has mainly focused on clinical or adult samples. To prevent suicides at an early stage, however, it is important to screen for risk factors in a community sample of adolescents. We compared the accuracy of logistic regressions, elastic net regressions, and gradient boosting machines in predicting suicide attempts by 17-year-olds in the Millennium Cohort Study (*N* = 7,347), combining a large set of self- and other-reported variables from different categories. Both machine learning algorithms outperformed logistic regressions and achieved similar balanced accuracies (.76 when using data 3 years before the self-reported lifetime suicide attempts and .85 when using data from the same measurement wave). We identified essential variables that should be considered when screening for suicidal behavior. Finally, we discuss the usefulness of complex machine learning models in suicide prediction.

According to the latest report of the World Health Organization (WHO, 2021) on adolescent mental health, suicide is the fourth leading cause of death in people aged 15 to 29 years worldwide. A recent cross-cultural meta-analysis including 686,672 children and adolescents estimated the lifetime prevalence for suicide attempts to be 6%, and for suicide ideation, 18% (Lim et al., 2019). Even at the early age of 9–10 years, 8.4% of 7,944 children interviewed in the US-based *Adolescent Brain and Cognitive Development* study reported having past or current suicide ideation and 1.3% confirmed attempted suicides (Janiri et al., 2020). Non-fatal self-harm and previous suicide attempts considerably increase the risk of subsequent suicide attempts (Beckman et al., 2018; Geulayov et al., 2019; Iorfino et al., 2020), making it crucial to better understand the factors associated with suicidal behaviors at an early age to prevent enhanced risk of deaths by suicides. However, previous efforts to predict suicide were often unsatisfactory, mainly because effect sizes of individual factors that have been shown to correlate with suicidal behaviors were only small to moderate (Franklin et al., 2017).

To address the multitude of small factors contributing to suicidal behavior, more recent studies argued for the use of advanced modeling techniques in the prediction of suicidal behaviors and thoughts (e.g., Fox et al., 2019; Huang et al., 2020). Machine learning (ML) algorithms can incorporate many and potentially collinear predictors and, therefore, constitute a useful tool for reflecting or condensing the complex processes including a myriad of factors and multiple phases that lead to suicide. Most previous studies using ML algorithms to predict suicidal behaviors included adult or clinical samples, whereas studies trying to predict suicide risk in non-clinical adolescent samples are rare (e.g., Bernert et al., 2020). However, for assessing the occurrence of and potentially preventing further escalations of adolescents’ suicidal behaviors, it would be pivotal to understand which factors are associated with suicidal behavior in the general population of adolescents rather than those in treatment or hospitalized due to self-harm (e.g., Beauchaine et al., 2019). With this study, we try to predict self-reported lifetime suicide attempts in 17-year-old adolescents using a representative community sample from the longitudinal *Millennium Cohort Study UK* (MCS; Joshi & Fitzsimons, 2016). Besides logistic regression as a baseline model, we used ML algorithms, namely elastic net regressions (e.g., Zou & Hastie, 2005) and gradient boosting machines (GBMs; Friedman, 2001) to integrate a large number of variables in our prediction model to account for the multitude of potential risk factors and compare classification accuracy and stability across different time intervals. Furthermore, we examine whether specific variable categories such as mental health, drug use, personality, and so forth are important for the prediction of lifetime suicide attempts, which would render them informative for public screening.

## Suicide as a Complex Interplay of Various Facilitating Factors and Acute Risk

In the following, we will discuss aspects impeding an accurate prediction (and potential prevention) of suicidal behaviors in adolescents: (a) the very heterogeneous pool of individual risk factors with small effects, (b) the low prevalence of suicide attempts and deaths by suicide, and (c) the difficulty in exactly pinpointing the timing of suicidal behaviors. Within a comprehensive meta-analysis on risk factors for suicidal thoughts and behaviors by Franklin et al. (2017), previous self-harm has been shown to be the strongest predictor of suicide attempts across all age groups. The authors summarized the research of the last 50 years, stating that individual effects of various predictor variables across 16 categories (including internal and external psychopathology, normative personality, demographics, physical illness or social factors) have been small, and the prediction of suicide attempts was unsatisfactory (with weighted area under the curve [AUC] values of .49–.61). Nevertheless, there is ample research including multiple meta-analyses on so-called warning signs or risk factors of suicidal behaviors. Factors that have been meta-analytically shown to enhance the risk of suicidal behaviors include accumulated childhood adversity (Björkenstam et al., 2017), perfectionism (Smith et al., 2018), sleep problems (Kearns et al., 2020), hopelessness and depression (Ribeiro et al., 2018), anxiety sensitivity (Stanley et al., 2018), as well as mental disorders in general (Too et al., 2019).

Hawton et al. (2012) assigned previously identified risk factors of self-harm and suicide in adolescents into three broad categories: sociodemographic and educational (e.g., sexual orientation or low socioeconomic status), negative life events and family adversity (e.g., parental death, parental mental disorder, or bullying), and psychiatric and psychological (e.g., low self-esteem, perfectionism, or hopelessness). Similar factors have been reported for suicides of adolescents occurring between April 2019 and April 2020 based on death reviews (i.e., professional mandatory data collection on causes of death of all children younger than 18 years in England), namely household functioning, mental and physical health, loss of or conflict with key relationships, risk-taking behavior, drug misuse, problems with the law, abuse and neglect, bullying, problems at school, social media and internet use, and sexual orientation/gender identity (National Child Mortality Database [NCMD], 2021). The combination of small effects and a low prevalence rate of suicides in children and adolescents (e.g., 1.8 per 100,000 in children aged 9–17 years in 2019 in England; NCMD, 2021) renders modeling suicide risk complicated, often resulting in low positive predictive values, that is, the proportion of true suicides or suicide attempts of all cases that are classified as such (e.g., Belsher et al., 2019). The prediction of suicide is further complicated by the low specificity of potential risk factors such as bullying, so that even among the group of adolescents affected by multiple risk factors, the vast majority will not attempt or die by suicide.

Another aspect worth considering in the prediction of suicide attempts in adolescents is that rates of suicidal behaviors differ across age within childhood and adolescence. For example, in England during the period of April 2019 to April 2020, 46% of all children’s and adolescents’ suicides occurred in 17-year-olds (compared to 16% in 15- and 16-year-olds and 22% in the group of 14-year-olds and all younger children). An overview of global deaths by suicide showed a similar trend (Naghavi et al., 2019). In 10- to 14-year-olds, the rate was 1.3 per 100,000. This number increases to 8.4 per 100,000 in 15- to 19-year-olds, demonstrating a sharp increase in self-harming and suicidal behaviors in the phase of later adolescence. Research on the developmental course of risk factors of suicide attempts suggests that while there are factors (e.g., previous self-harm, psychological distress) that enhance suicide risk across age groups, there may also be factors that are particularly relevant for specific age groups. For example, Lear et al. (2020) examined whether risk factors for suicide ideation and suicide attempts differed between middle school (aged 11–14 years) and high school students (aged 14–18 years) and found that general psychological distress was associated with suicide attempts in both groups, whereas feeling unsafe at school, lacking family involvement, and community disorganization were only significantly associated with suicide attempts in middle school students. The authors propose a higher dependence on family or other adults in general for middle school students as an explanation for this finding.

## Predicting Suicidal Behaviors Using Machine Learning Algorithms

The usefulness of ML algorithms in suicide research is controversial (e.g., Siddaway et al., 2020). On one hand, there is evidence for an enhanced predictive accuracy by ML models on suicide compared to less complex models such as logistic regressions (Burke et al., 2019). In addition, a recent meta-analysis encourages the notion that ML algorithms outperformed theory-driven predictions of suicide ideation, attempts, and deaths by suicide in longitudinal studies (Schafer et al., 2021). On the other hand, although statistical predictions overall slightly outperform clinical predictions (e.g., Ægisdóttir et al., 2006; Grove et al., 2000), this increased predictive accuracy comes at the cost of lower interpretability. Moreover, the fact that positive predictive values are still rather low, even in models using high-risk, clinical samples (Belsher et al., 2019; Kessler et al., 2019), raises questions about their practical usability. In addition, some researchers emphasized the methodological pitfalls that can occur in estimating complex models including non-linear and interaction effects, possibly leading to biased results (e.g., Jacobucci et al., 2021). In summary, ML algorithms might be useful for regularization in data-driven analyses when there is a plethora of (intertwined) variables, but it is necessary to ensure the generalizability of the ML-based results.

Table 1 provides an overview of five recent studies that use ML algorithms to predict suicide attempts in community samples including adolescents or young adults. Some characteristics of these studies make it difficult to compare and summarize the results. First, the studies operationalize the outcome “suicide attempt” differently in terms of time frame (e.g., last week, last 12 months, or lifetime) or whether attempts are combined with suicidal thoughts. Second, although we solely included studies with adolescent or young adult samples, age varies widely within studies (up to 16 years) and across studies. Third, different methodological approaches regarding model validation, handling of missing data, and handling of unequal group sizes further complicate an unbiased comparison. Bearing these constraints in mind, one main finding across all five studies was the importance of previous suicidal thoughts, suicide attempts, or self-harm when predicting suicidal behaviors, which is in line with recent meta-analyses (e.g., Franklin et al., 2017). Variables reported as important predictors beyond previous self-harm largely depended on the settings of the respective study. For example, van Mens et al. (2020) stated that their variable set was limited insofar as it only comprises psychological risk factors although demographics, lifestyle behaviors, or victimization have been shown to be influential in the other four studies.

**Table 1:** Overview of Studies Predicting Suicide Using Machine Learning in Adolescents and Young Adults.

Study	Sample	Outcome and prevalence	Design and method	Predictor variables	Predictive accuracy	Important predictors
Burke et al. (2020)	Primary care patients from Pennsylvania (*N* = 13,325) between 14 and 24 years of age (*M*_age_ = 17.06, *SD*_age_ = 2.61)	(a) Suicide attempt in the last week (*n* = 39, 0.3%) (b) Lifetime suicide attempt (*n* = 608, 4.6%)	Cross-sectional, ridge regressions and random forests, 200 imputed data sets split into training (75%) and testing (25%) samples	107 Variables covering demographics, school, family, safety, substance use, nutrition, safety, sexual risk, medical history, depression, anxiety, psychosis, trauma, bullying, gun access	Random forest: .84 (lifetime) .76 (last week) Ridge regressions: .87 (lifetime) .77 (last week)	Active and passive suicidal ideation, suicide planning, and non-suicidal self-injury, physical abuse
Hill et al. (2019)	National (USA) Longitudinal Study of Adolescent to Adult Health (*N* = 4,834), *M*_age_ = 16.15, *SD*_age_ = 1.63 at wave 1	Number of attempted suicides in the past 12 months, coded 0 for 0 and 1 for > 0 (*n* = 192, 3.97%)	Longitudinal, classification trees, no validation using independent test data	345 Variables covering mental health, victimization, negative life events, family, peer and school functioning, and community engagement	Results of different classification trees are presented, tree 15 with the highest accuracy: .80	Past suicide ideation, depression, mother’s education and work, risky behaviors, sexual behavior and sexually transmitted diseases, substance use, expectations about romantic relationships
Macalli et al. (2021)	French i-Share cohort, volunteer student sample (*N* = 5,066), *M*_age_ = 20.7, *SD*_age_ = 2.6	Participants having occasional or frequent suicidal thoughts and/or reported suicide attempts (*n* = 874, 17.3%)	Longitudinal, random forests, 10-fold cross-validation, no validation using independent test data	70 Variables covering demographics, lifestyle, family, physical health, substance use, psychiatric disorders, lifetime suicide attempts, suicidal thoughts in the last 12 months, depression, anxiety, self-esteem, perceived stress, and impulsivity	AUC of .84 for girls, .82 for boys	Suicidal thoughts at baseline, self-esteem, trait anxiety, and depression symptoms
van Mens et al. (2020)	Scottish wellbeing study (*N* = 3,508) between 18 and 34 years old (*M*_age_ = 20.7, *SD*_age_ = 2.6)	Participants reported a suicide attempt in the last 12 months (*n* = 50, 2.0%)	Longitudinal, multiple machine learning algorithms, dataset split once into training (70%) and testing (30%) samples, 10 × 10-fold cross-validation	211 Items assessing psychological risk factors such as depression, stress, wellbeing, defeat, entrapment, social support, interpersonal needs, goal activation, optimism, resilience, acquired capability, impulsivity, death-related mental imagery, and history of suicidal ideation and suicide attempts	Logistic regression: .51, *k*-nearest neighbors: .64, classification tree: .71, random forest: .66, gradient boosting: .69, support vector machine: .65	Acquired capability, defeat, depressive symptoms, and a history of suicide attempts
Van Vuuren et al. (2021)	Dutch community sample (*N* = 8,998) Students in the 2nd and 4th year of secondary education (13- to 14-year-olds and 15- to 16-year-olds)	Ask Suicide-Screening Questionnaire—Revised, coded as 1 if any of 4 questions (on recent suicide ideation and lifetime attempts) were affirmed (*n* = 732, 8.3%)	Longitudinal, comparison of random forest and LASSO algorithms to a decision rule that classifies every student as “at risk” who affirmed suicidal behaviors at baseline, dataset split once into training (70%) and testing (30%) samples, 10 × 10-fold cross-validation	Demographics, lifestyle behaviors, physical and mental health, (un)safe environment, and whether made use of information or help on the provided websites or from a school nurse	Decision rule: .64 random forest: .65 LASSO: .68	Suicide-screening score at baseline, nutrition, drug abuse

*Note.* All samples are community samples. For Burke et al. (2020), we only present the results of primary care patients (not emergency care patients). Unless otherwise stated, accuracy values are balanced accuracies. If balanced accuracy values were not reported within a study, we calculated them (mean of specificity and sensitivity). For studies with multiple outcomes, we present accuracy values referring to the outcome that includes suicide attempts. Important predictors are presented for the most accurate model predicting suicide attempts.

AUC = area under the curve; LASSO = least absolute shrinkage and selection operator.

Predictive accuracies varied across studies, modeling approaches, and outcome type (e.g., balanced accuracies between .51 and .87), and there was no clear picture as to whether more complex algorithms that allow for non-linear or interaction effects (e.g., random forest or gradient boosting) necessarily lead to more accurate predictions than (regularized) linear regressions. This was often due to a lack of explicit comparison between the respective ML algorithms and simpler approaches. Another methodological shortcoming of four out of five studies presented in Table 1 concerns the lack of independent or unbiased model validation. The most rigorous way of quantifying the predictive accuracy of a model–validation with independent data of an entirely new study (Dwyer et al., 2018)—was not used in any of the studies. In three of the five studies, the full data were split into training and testing data sets, a less optimal but still much-used strategy in ML (e.g., Christodoulou et al., 2019). However, in two of those studies, the authors divided their sample only once. In these cases, ML models tend to fit to the noise in the training data, especially when the features-sample size ratio is high (e.g., Vabalas et al., 2019). Thus, predictive performance and regression coefficients might unduly depend on chance, that is, on which persons were sampled into training or testing data. To minimize this bias and obtain a more robust estimate of predictive accuracy, the procedure needs to be repeated many times.

## The Present Study

In this study, we aim to predict self-reported lifetime suicide attempts by 17-year-old adolescents using data from the longitudinal MCS. We rely on a large set of predictor variables from self-reports and other reports by adolescents and their families covering 14 categories including physical and mental health, drug use, victimization, personality, or future goals (see Table 2). Generally, we use the term *predict* in a statistical fashion, that is, as a statistical abstraction of the relation between a set of variables (predictor variables) and an outcome. We do not imply a causal relationship or a strict temporal order.^1^ This study pursues three goals.

**Table 2:** Predictor Categories With Example Items.

Category	Topics of example items	Number of predictors	
		Model 1/2	Model 3
Activities	Going to the cinema	24	31
Attitudes	Attitudes concerning gender equality	12	19
Behavior	Misbehaving in lessons	22	18
Demographics	Current legal marital status	37	39
Drug use	Smoking e-cigarettes	16	22
Emotion & motivation	Hating oneself	34	40
Family	Parent working long hours	50	48
Future goals	Estimated likelihood of attending university	3	12
Mental health	Currently treated for depression or anxiety	16	38
Offenses (illegal)	Ever been arrested	21	27
Personality	Conscientiousness	39	83
Physical health	Having diabetes	59	58
Sexuality	Having had sexual intercourse with another young person	17	20
Victimization	Being hurt or picked on by other children	7	17

First, we investigated to what extent it is possible to predict self-reported lifetime suicide attempts using ML algorithms. In doing so, we compared the predictive accuracy of elastic net regressions and GBM with logistic regressions. The overarching goal of regularized regressions is to avoid overfitting by penalizing too complex models (e.g., Cox et al., 2020). Elastic net regression finds a compromise between least absolute shrinkage and selection operator and ridge regressions to strike a balance between minimizing the sum of squared weights (assigning variables small but non-zero weights) and the sum of absolute weights (leading to models with many variables given weights of zero). In contrast to logistic regressions and elastic net regressions, GBM algorithms allow for the integration of non-linear and interaction effects without making a priori assumptions on specific functions between predictor variables and the outcome (James et al., 2017; Schroeders et al., 2022). When predicting suicidal behaviors, many potential moderators are conceivable. King et al. (2014), for example, advocated the necessity of compiling and validating suicide screenings separated by gender because they only found a relation for suicide ideation and subsequent suicide attempts within a year after hospitalization for girls. In addition, initial findings from the MCS suggested that there are inequalities in the prevalence of psychological distress and suicidal behaviors across gender, ethnicity, sexual orientation, and socioeconomic status for 17-year-old adolescents (Patalay & Fitzsimons, 2020). In this report, differences between gender groups and sexual orientation were highlighted: More than twice as many girls than boys confirmed a previous suicide attempt (10.6% vs. 4.3%), and for LGB+ adolescents, the rate was even higher (21.7%). It, therefore, seems worthwhile to incorporate these and other potential moderating effects into the prediction of suicidal behaviors in adolescents.

Second, we also investigated if it is possible to predict lifetime suicide attempts 3 years before they were reported. Although there was no information available on the exact timing of the lifetime suicide attempts, given the prevalences described above, we assume that the large majority of suicide attempts occurred after the adolescents turned 14 years of age. For the prediction of future suicide attempts based on electronic health records, Walsh et al. (2017) found that accuracy improved as the assessment of predictors and suicide attempts was closer in time. It is likely that this finding generalizes to other longitudinal studies such as the MCS. Thus, we also examined the extent to which predictions get more accurate when predictors and outcome were assessed at the same measurement wave as compared to 3 years ahead of time.

Third, we provided an overview of the relative variable importances across models and discussed potential patterns or groupings of predictors of suicide attempts that could inform future theory-building or systematic screenings. We also scrutinized if variable importances shift between models using different variable sets and time frames. Using a longitudinal data set, we avoided typical problems of cross-sectional data, that is, potential changes in the variable importance would indicate that models on suicide need to account for the specific developmental stages of children and adolescents.

This study was not preregistered. All analyses are exploratory; we had no specific prior hypotheses regarding the three aforementioned goals or research questions apart from the respective rationales we presented (see also Wagenmakers et al., 2012).

## Method

### Sample

The MCS UK (Joshi & Fitzsimons, 2016) is a longitudinal cohort study comprising 18,818 children born in the United Kingdom in 2000–2001. Participants were sampled from clusters of electoral wards, with ethnic minorities being disproportionally stratified. We used data from the sixth (conducted in 2015, *n* = 11,872) and seventh (conducted in 2018; *n* = 10,757) measurement waves in which the participants were 14 and 17 years old, respectively. Both measurement waves have been approved by research ethics committees (ref 13/LO/1786 and 17/NE/0341). We used all cases for which the adolescents’ interview at the seventh measurement wave was available (*n* = 10,345), included only one child per family (*n* = 10,238), and discarded all cases in which the outcome variable (“Have you ever hurt yourself on purpose in an attempt to end your life?”) was missing (resulting in *n* = 9,723). Finally, we only included adolescents whose self-reports and other reports (in 99.4% reported by one of the parents) were available in both Waves 6 and 7 to avoid issues of attrition (Jankowsky & Schroeders, 2022). This resulted in a total of *n* = 7,347 participants. To check whether there were systematic differences between these 7,347 and the excluded cases, we compared the correlations of all variables in the adolescents’ interview to the outcome across both disjoint samples. The averaged absolute difference between person correlation coefficients was .03 (*SD* = .02). Absolute differences ranged from 0 to .31 with a median of .02. About 83% of the correlation differences across samples were smaller than .05%, and 99% were smaller than .10, which is often used as a rough upper limit for small effect sizes (Gignac & Szodorai, 2016).

The gender ratio of this subsample was balanced; 3,571 (48.6%) of the adolescents were male. Families with higher incomes were overrepresented within the sample: In the sixth measurement wave, 10.6% of the families were categorized in the lowest income quantile, 13.9% in the second-lowest, 19.3% in the middle, 26.4% in the second-highest, and 29.7% in the highest. The majority of adolescents (86.3%) were White, 1.0% were of mixed race, 2.6% were Indian, 3.8% were Pakistani, 1.4% were Bangladeshi, 1.0% were Black Caribbean, 1.7% were Black African, and the remaining 1.9% were summarized into an “other” category. For a more detailed overview on participants’ demography, please see Supplemental Table S1.

### Measures

We selected a broad range of variables to predict self-reported lifetime suicide attempts (overall *N* = 638). For an overview, we present the categorization scheme used to classify the predictor variables, together with some example items and the overall number of predictors in each category (Table 2). We used the raw data at item level wherever available to fully capture any potential item effects in suicide prediction (e.g., McClure et al., 2021). We dummy-coded all categorical variables before the analysis using the first category as reference. The outcome measure we used in the present analysis was a single variable of the seventh wave assessing lifetime suicide attempts (“Have you ever hurt yourself on purpose in an attempt to end your life?”) coded as 0 for *no* and 1 for *yes*.

### Statistical Analyses

We compared three different models for the prediction of lifetime suicide attempts: The first included 357 variables from the sixth survey wave of the MCS in which adolescents were 14 years old. Of these variables, 49.02% were answered by the adolescents’ parents, of which 42.86% were other reports about their children. In the second model, we updated wherever possible the information of the seventh survey wave in which the adolescents were 17 years old (i.e., for 153 of the 357 variables of the first model or 42.86%). In case a variable was not surveyed again, the original values of the sixth wave were taken. In the seventh survey wave, some variables previously answered by parents were answered by the adolescents so that the share of variables answered by parents decreased to 43.31%, out of which 35.57% were other reports. The third model comprised the same variables as the second model supplemented with 139 variables that were only available within the seventh wave (which were all self-reports by adolescents, decreasing the share of variables answered by parents within the third model to overall 31.57%). These newly available variables predominantly fall into the categories future goals, mental health, personality, and victimization (see also Table 2). By including both the second and the third models in our analyses, we were able to disentangle the effects of time (i.e., more current information likely being more predictive assuming the respective suicide attempts were recent) and content (i.e., incremental validity of newly added variables). All analyses were conducted using the R package caret (Kuhn, 2008) as a wrapper interface for modeling and prediction. We compared the predictive accuracy of logistic regressions, elastic net regressions (using the package glmnet; Friedman et al., 2001), and GBM (using the package gbm; Greenwell et al., 2019). All supplemental materials including analyses scripts, supplemental figures, and a list of all categorized variables are available at https://osf.io/bycvd/. The data reported in this manuscript are publicly available data from the MCS and can be accessed after registration with the UK Data Service.

For an unbiased model evaluation, we split the full data into a training data set (80%) and an independent testing data set (20%). Missing values were imputed separately for the training and testing data sets (i.e., after the 80/20 split) using the *k*-nearest neighbors algorithm implemented in caret. For most of the variables (about 80%), the amount of missingness was small (<5%), and the average missingness was 4% across all variable sets. For model training, we used 10-fold cross-validation with upsampling, which means that persons from the minority group (i.e., suicide attempters) were upsampled to match the size of the non-attempters in the training data set. Upsampling is a common and robust procedure often used to handle imbalanced data sets (e.g., García et al., 2012). Parameters for the elastic net regressions (the shrinkage parameter λ and the penalty parameter α; Zou & Hastie, 2005) were tuned using a tuning length of 21. GBM are tree-based ML algorithms that sequentially combine multiple decision trees, also called “weak learner,” into an ensemble. Every new tree aims at fitting the residual error of the previous one, leading to a potentially better predictive performance. However, they also run the risk of overfitting, which should be counteracted with sensible hyperparameter tuning (e.g., McNamara et al., 2022). For the gbm tuning parameters, we used the following settings: interaction depth of 1, 2, 3, or 4; a minimum leaf size of 5, 10, 20, or 50; a sequence of shrinkage values between .051 and .201 using steps of .01; and five different numbers of trees (50, 100, 150, 300, and 500).

To evaluate the classification into adolescents who ever attempted suicide vs. adolescents who never attempted suicide, we report the balanced accuracy (the mean of sensitivity and specificity), sensitivity, specificity, and the positive predictive value. In our analyses, sensitivity represents the ratio of correctly identified attempters to all attempters. Specificity represents the ratio of correctly identified non-attempters to all non-attempters, and the positive predictive value represents the proportion of true attempters out of all adolescents who were flagged as attempters. All indices were calculated for each testing data set across 100 iterations of splitting the data into training and testing data.

## Results

Overall, 502 of the 7,347 seventeen-year-old participants (6.83%) indicated that they had hurt themselves on purpose in an attempt to end their life at some point in their life. In the following paragraphs, we first present how accurately this outcome could be predicted by the three different models described above. In a subsequent step, we examine which variables predict these self-reported lifetime suicide attempts and whether the set of the most predictive variables varied across models.

**Figure 1:**
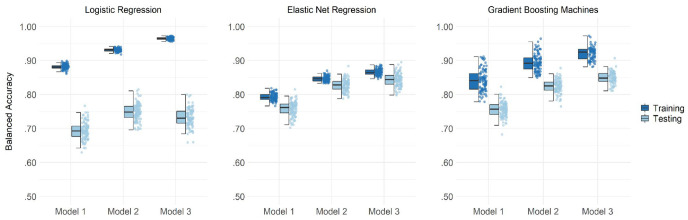
Balanced Accuracies for Predicting Suicide Attempts in Adolescents *Note*. The boxplot reflects the interquartile range, the solid line represents the median, and the whiskers 1.5-times the interquartile range of 100 iterations. Balanced accuracies are displayed as jittered distribution on the right.

Figure 1 shows the balanced accuracies of 100 iterations of logistic regressions, elastic net regressions, and GBM for three models. The first one used data from the sixth wave of the MCS including 14-year-olds (Model 1: 14 years). In the second model (Model 2: 14 years updated), all variables of the first model were updated if the information was available in the seventh wave, otherwise the original variable was kept in the data set. Finally, the third model (Model 3: 17 years) used variables of the seventh wave that were not available in the previous assessment in addition to the variables of Model 2. Considering only the predictive accuracy within the testing data sets across all models, elastic net regressions and GBM models achieved similar averaged balanced accuracies (ABAs) (.76 and .76 for Model 1, .83 and .82 for Model 2, and .84 and .85 for Model 3, respectively), whereas the predictions with logistic regressions were clearly less accurate (ABA of .69 for Model 1, .75 for Model 2, and .73 for Model 3). This can be explained by the fact that, even with a relatively large sample (with a testing sample of 1,470 adolescents), the logistic regressions were highly overfitted with differences of .19, .18, and .23 in ABA between training and testing (see Figure 1, panel A). Overfit was less pronounced for the GBM models (differences of .07 for all models; Figure 1, panel C) and smallest for the elastic net regressions (differences of .03, .02, and .03; Figure 1, panel B), showing that both ML algorithms efficiently used regularization to handle the large number of predictor variables.

Irrespective of the specific algorithm, the above-described ABAs also show that predictions were more accurate for models using variables that were assessed at the same time as the self-report information on lifetime suicide attempts than for models that relied on data from the 14-year-olds only. There was only a negligible difference in the averaged predictive accuracy between Model 2 and Model 3 which both used data from the 17-year-olds. Temporal proximal predictors thus led to higher accuracies than distal ones (again, assuming that the lifetime suicide attempts were more closer in time to the second measurement wave we used in this study). Adding variables that were only available in the assessment of the 17-year-olds—predominantly variables about future goals, mental health, personality, and victimization—had overall little incremental predictive value.

**Figure 2:**
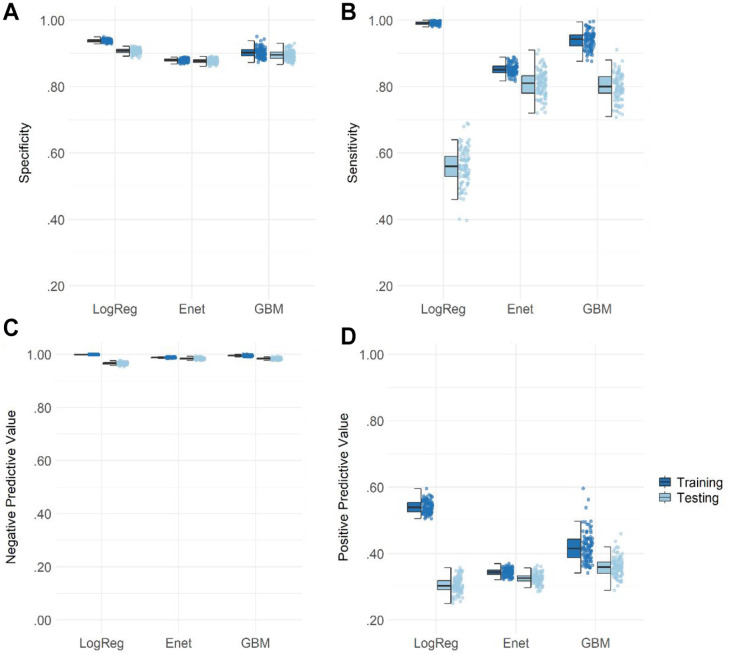
Specificity, Sensitivity, and Negative and Positive Predictive Values for Predicting Suicide Attempts in Adolescents in Model 3 *Note*. LogReg = logistic regression, Enet = elastic net regression, GBM = gradient boosting machines. The boxplot reflects the interquartile range, the solid line represents the median, and the whiskers 1.5-times the interquartile range. Specificities (A), sensitivities (B), negative predictive values (C), and positive predictive values (D) are displayed as jittered distribution on the right.

Figure 2 shows a comparison of the specificities, sensitivities, negative predictive values, and positive predictive values across the three modeling algorithms for Model 3 (for a similar overview of Models 1 and 2, see Figure S1 and S2). Averaged specificities in the testing data set (.91 for logistic regression, .88 for elastic net regression, and .90 for GBM) were generally higher than sensitivities (.56 for logistic regression, .81 for elastic net regressions, and .80 for GBM), meaning that the group of non-attempters could be detected more accurately than those who did report suicide attempts irrespective of the modeling approach. Although there were minor differences in specificity between algorithms, sensitivity was much lower for the logistic regression. Averaged negative predictive values were high irrespective of modeling algorithm (.97 for logistic regression, .97 for elastic net regressions, and .98 for GBM), indicating that nearly all who were classified as not at risk were correctly identified as such. The average positive predictive value or precision was lowest for the logistic regressions (.30), higher for the elastic net regressions (.33), and highest for GBM (.36), indicating that, even in the best model, only about one third of all flagged respondents is correctly identified as suicidal (i.e., the flagging was false in two thirds of the cases).

To sum up, using all available variables for 17-year-olds, it was possible to detect over 80% of adolescents who ever attempted suicide. Moreover, there were no significant differences in predictive accuracy between the elastic net models and GBM models in our study, contradicting earlier findings in suicide prediction. Because the training model of the elastic net regressions showed the least overfit (or in other words, is likely most transferable to unseen data) and tree-based ensemble models tend to be less straightforward to interpret (Cox et al., 2020), in the following paragraphs, we will focus on the variable importance of the elastic net regressions. However, we will also present variable importance of the GBM models for comparison and as a robustness check of our results.

### Important Variables in Predicting Suicide Attempts

**Figure 3:**
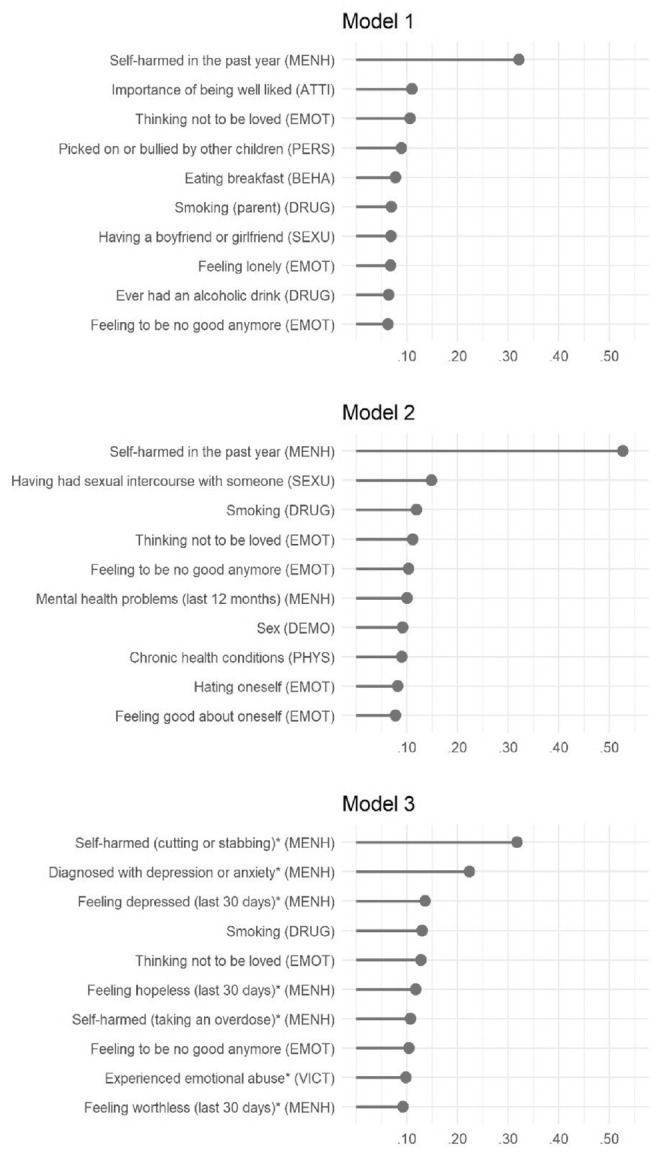
Averaged Standardized Regression Coefficients as Indicators of Variable Importance *Note.* The variable’s category is given in parentheses following the respective label. MENH = mental health; EMOT = emotion and motivation; ATTI = attitudes; PERS = personality; BEHA = behavior; DRUG = drug use; DEMO = demographics; SEXU = sexuality; PHYS = physical health; VICT = victimization. The asterisk (*) denotes newly added variables in Model 3.

In Figure 3, we show the 10 largest averaged standardized regression coefficients of the different elastic net regressions to indicate their importance in the prediction (for the 50 most important predictor variables per model, see Figure S3–S5). Because the differences between the effects of individual variables were often small, the rank order should not be given too much weight. Nonetheless, we provide a short overview of which categories were most predictive. In the following paragraphs, we will summarize three major points: First, the most important variables across the three models by far were indicators of previous self-harm. The question arises as to what extent algorithms that can incorporate several hundreds of variables have incremental value over a simple decision rule that classifies every adolescent who ever showed previous self-harming behavior as “at risk” (e.g., Van Vuuren et al., 2021). Using such a single-item decision rule (i.e., classifying every 17-year-old who confirmed previous self-harm at 14 years of age as “at risk”), balanced accuracy was only slightly lower than that in the first model (.74 vs. .76), but sensitivity was substantially lower (.59 vs .69). Thus, in our study, such a simplified decision rule would be less sensitive than using ML algorithms with all information available.

Second, if we categorize the most important predictors across all models, these were (in descending order), mental health, emotion and motivation, drug use, sexuality, demography, victimization, physical health, personality, attitudes, and behavior. Out of the category emotion and motivation, “thinking not to be loved” and “feeling to be no good anymore” as indicators for loneliness and low self-esteem were among the 10 most important predictors irrespective of model. Of the 30 most important predictors (10 per model), all but two variables of the first model (“smoking” and “bullying”) were self-reports by adolescents. Third, for some variables, a shift in importances across the three models can be detected, that is, different categories of variables were specifically important for the prediction of lifetime suicide attempts at a specific point in time. For example, in Model 3, variables of the categories mental health and emotion and motivation were among the 10 most important predictors, while these variables were not as important at an earlier developmental stage. This shift can probably be explained by the more fine-grained and reliable assessment of self-harm (cutting or stabbing and taking an overdose were ranked first and third) and the inclusion of the six items of the Kessler Psychological Distress Scale (Kessler et al., 2002) in the assessment of the 17-year-olds (see the variables asking about feelings within the last 30 days in Figure 3). Looking at the 50 most important variables in each model (for an overview, see Supplemental Figures S3–S5), some variables of the categories sexuality, drug use, illegal offenses, or victimization also tended to gain significance across adolescence. It should be noted that this is also due to the fact that the prevalences of these behaviors significantly increase within the given age range (e.g., sexual intercourse with a peer).

Supplemental Figures S6–S9 show the 10 or 50 most important variables for the GBM models. Overall, the overlap in important variables was large across modeling approaches: Out of the 10 most important variables of the elastic net models, 60% (Model 1), 70% (Model 2), and 90% (Model 3) were also among the 10 most important variables of the GBM models, and all those 10 (for all models) were among the 50 most important variables of the GBM models. Regarding the 50 most important variables, overlap between elastic net and GBM models was at 68% (Model 1), 80% (Model 2), and 70% (Model 3), with more deviations for lesser important variables (which can be expected due to the very small differences in effects and, thus, unstable rank order among the lesser important variables). All in all, the conclusions we derived of the elastic net results about important variable categories equally apply to the GBM models.

## Discussion

Every death by suicide in adolescence is a tragedy. Being able to accurately model and better understand suicidal behaviors in adolescents is literally a vital goal as factors relevant in predicting lifetime suicide attempts of 17-year-olds could also be relevant for preventive screening tools when implemented at earlier ages. Beauchaine et al. (2019, p. 643) argued for interventions to take place even in childhood since “early starters exhibit greater frequency of non-suicidal self-injury, use more diverse and dangerous methods, and are hospitalized more often than later starters.” Screenings with clear-cut decision rules such as the Oxford Mental Illness and Suicide Tool (Fazel et al., 2019) have been specifically developed for adult and clinical samples. However, they mainly address samples with severe mental disorders (e.g., schizophrenia spectrum or bipolar disorder), and they usually require some clinical expertise. In this study, we tried to narrow down the range of potentially relevant variables in predicting lifetime suicide attempts in 17-year-olds in the United Kingdom using self-reports and other reports as typically administered in large-scale panel studies. Generally, the predictive accuracy in the current investigation was higher than that in most other similar studies analyzing adolescent community samples (see Table 1 for a point of comparison). Overall, we evidenced that it is possible to accurately model lifetime suicide attempts using data from longitudinal (household) panels although the variables were not specifically included for this purpose (i.e., for assessing constructs known or hypothesized to affect suicidal behaviors, such as in van Mens et al., 2020). Results of studies like ours can provide valuable information on what variables might enhance lifetime suicide attempts screenings for adolescents.

In any classification task, researchers have to decide whether to consider sensitivity and specificity equally—as we did in the present study—or to optimize one metric at the cost of the other. Regarding suicide attempts by adolescents, undoubtedly the false negative rate (= miss rate) should be as low as possible, which is equal to having a high sensitivity. Overall, we found that non-attempters could be predicted more accurately than attempters, that is, specificity was higher than sensitivity for all models. Adding new variables from the assessment of the 17-years-olds (Model 2 vs. Model 3) did not significantly change sensitivity but led to higher specificity, thus reducing the number of false alarms. Whether false alarms are problematic depends on the consequences that will be drawn from the modeling. For instance, low-cost brief-contact interventions or information materials could easily be provided broadly to adolescents with a high risk score, and false alarms might pose less of an issue in these low-threshold offers. However, it has been meta-analytically shown that brief-contact interventions only slightly reduced the overall number of repeated self-harm incidents per person and not the odds of death by suicide (Milner et al., 2015). In contrast, more extensive (and possibly more effective) interventions such as individual cognitive therapies (Zalsman et al., 2016) are more expensive and are only available to some individuals. To use personnel and financial resources adequately, it is thus essential to offer help to the most vulnerable adolescents, which is analogous to high specificity and a positive predictive value. On a side note, if researchers were to inform parents of each adolescent with a (very) high risk score, false alarms could also lead to additional burden on adolescents and parents, irritation, or subsequent underreporting of suicidal behaviors (see Kleiman et al., 2019 for a similar argument in real-time monitoring).

### The How and the When of Suicide Screening

In the present analyses, our prediction could not draw upon data of a strict temporal order. Rather we modeled any adolescents’ suicide attempts (depending on the model either fully or partly past) and examined variable categories that were especially informative, making them promising candidates for the inclusion in screening instruments for overall lifetime suicide risk. Our results are in line with previous research on suicide risk (e.g., Franklin et al., 2017; Hawton et al., 2012), with the most informative variables including previous self-harm, mental and physical health problems, victimization, lack of future goals, drug misuse, atypical or negative sexual experiences (in relation to a specific developmental phase), and psychological constructs covering distress including feelings of hopelessness, loneliness, thwarted belongingness, and low self-esteem. Reassuringly, the predictors that were important in our study are overall similar to those already included in established tools for the assessment of suicide risk in adolescents (such as the Tool for Assessment of Suicide Risk Adolescent Version Modified; Kutcher, 2013), but our results also offer some pointers as to how screenings for suicide risk in adolescents could be improved. Broad screening tools differ from typical suicide risk assessment: Risk assessments in mental health facilities are often filled out by clinicians. Moreover, the evaluation as to whether, for example, an adolescent shows signs of anger or impulsivity is often done with a two- or three-level clinical rating scale. In contrast, we would recommend the inclusion of a more fine-grained and often continuous assessment of risk factors in self-report screening instruments. Furthermore, including (more) open questions about specific self-harming behaviors in broad screening instruments might also add valuable information as we found that different self-harming behaviors were associated with different risks of suicide attempts: Variables such as cutting or stabbing oneself or taking an overdose of pills were more informative than, for example, burning or bruising oneself. In line with this, Beckman et al. (2018) also showed different odds for subsequent suicide attempts depending on previous self-harming behaviors (i.e., more “violent” methods such as hanging, drowning, or jumping from a height led to higher subsequent suicidal risk). In addition, the frequency of self-harming behaviors should also be assessed as Ammermann et al. (2017) found differences in relations to psychopathology symptomology between individuals that self-harmed more severely (i.e., more often) and those who report five or less self-harming acts, even among individuals who engaged in self-harming behaviors at least once.

The results of the present analyses are also interesting with respect to what was not found: Apart from the mental and physical health of parents, information given by or about members of the adolescents’ family played only a negligible role in the prediction of adolescents’ lifetime suicide attempts. The information provided through other reports is thus not decisive in modeling lifetime suicide attempts in adolescents, which is in line with DeVille et al. (2020) who showed that caregivers underreport suicidality and self-harming behavior in their children: Eighty-eight percent of the caregivers had no knowledge that their 9- to 10-year-olds reported previous suicide attempts. Thus, although self-reports per se are prone to typical biases such as impression management, they are often a better source of information when it comes to internal or self-evaluative processes, especially in the absence of additional hard facts such as clinical records. Previous studies on adults repeatedly showed that diagnosed mental disorders are prominent risk factors of suicide (e.g., Cavanagh et al., 2003), which was not the case for the present sample of 14-year-olds. A possible explanation is that mental disorders are usually not diagnosed at this young age, a situation which especially holds true for the 14-year-olds and might change in the future with the *Diagnostic and Statistical Manual of Mental Disorders, fifth edition*, and a stronger developmental perspective of mental disorders (Clark et al., 2017). But it also shows that the significance of the predictor sets varies with age. Accordingly, preventions should be tailored to specific phases of development. Although there were many similarities in the variable importances across models, specific variables on sexuality, victimization, offenses, or drug misuse were more important for the 17-year-olds or only available for 17-year-olds. These behaviors have largely different prevalence rates and, therefore, different meanings for 14-year-olds compared to 17-year-olds. Thus, it might be promising to include questions that predominantly concern older adolescents (from a normative view) in the assessment of younger adolescents because they might point to an unusual or problematic behavior.

### Model Complexity Does Not Revolutionize Suicide Prediction

A frequently highlighted advantage of tree-based ML algorithms is the possibility of including non-linear effects or interactions into prediction models without the need for specifying a priori theoretical assumptions about specific predictor variables and their relation to the respective outcome. The idea that these algorithms could lead to an increment in the prediction of suicide behaviors is compelling and has already been put forward by several recent studies (e.g., Fox et al., 2019; Walsh et al., 2017). In our study, both ML algorithms that use regularization (and are thus better equipped to handle overfitting) achieved a higher predictive accuracy than a simple logistic regression with all predictors. Our results, thus, also emphasize that ML algorithms can enhance accuracy in predicting suicide attempts. However, using models that are capable of including non-linear effects or higher-order interactions such as GBM did not result in a more accurate prediction.

The lacking superiority of more complex models might be attributed to different factors: First, we used a model validation approach that strictly separates the training data from an independent holdout testing data, preventing information leakage between both data sets. Jacobucci et al. (2021) convincingly demonstrated that the higher predictive accuracies for suicidal behaviors in tree-based algorithms such as random forests that have previously been reported in the literature were largely based on a specific validation approach called “optimism bootstrapping” which can lead to inflated predictive accuracies. In other words, previous results demonstrating an advantage for more complex models in suicide prediction should be treated cautiously, and presumably the relations between predictor variables and suicide attempts are mostly linear. Second, simulation studies showed a measurement error can impact the ability of tree-based algorithm to accurately depict the non-linear effects or interactions contained in the true model of simulated data. Accordingly, GBM models did not achieve a higher prediction accuracy than linear regressions when the measurement error was high (e.g., Jacobucci & Grimm, 2020; McNamara et al., 2022). Given that we used many “fuzzy” psychological predictors that are affected by measurement error (e.g., indicators of emotions, feeling, or personality), similar issues may have occurred in our data.

At first glance, our results might seem discouraging for researchers aiming to use more-complex ML algorithms to further the prediction of suicidal behaviors. In fact, they only show that more complex models are no silver bullet that will always guarantee enhanced predictive accuracy but that “human” tasks such as trying to reliably assess constructs of interest, conducting appropriate model validation, or selecting relevant predictors are still important decisions for the researcher.

### Limitations

We would like to discuss two limitations of our study regarding the outcome variable, namely the single self-reported indicator of lifetime suicide attempts (“Have you ever hurt yourself on purpose in an attempt to end your life?”). The first limitation concerns the fact that lifetime suicide attempts were solely assessed for the 17-year-olds without an indication of the exact time point. Thus, it is not possible to rule out that some of these self-reported suicide attempts happened even before the first measurement wave we used for prediction, that is, when participants were younger than 14 years. However, suicide attempts become much more common at later stages of adolescence (e.g., Naghavi et al., 2019). For example, between 2019 and 2020, 78% of all adolescents’ deaths by suicide in England occurred in 15- to 17-year-olds; thus, the majority of attempts in childhood and adulthood will have occurred between the ages of 14 and 17 years. Although Models 2 and 3 (which use information only available after the respective suicide attempts) clearly predict past suicide attempts, we assume that Model 1 (which uses information from the assessment of the 14-year-olds) largely predicts future suicides. In addition, even predicting or rather modeling past suicide attempts in adolescents can be a worthwhile goal on its own and useful for prevention as it is a robust finding that previous self-harming and suicidal behavior is the strongest predictor of subsequent suicidal behaviors and, thus, enhances the risk of eventual deaths by suicide.

The second limitation refers to using a single self-report item. Hom et al. (2016) found that while all participants of their study sample endorsed a previous suicide attempt on a single-item self-report, only 60% had an actual suicide attempt history when a multi-item assessment and an in-person interview were conducted at follow-up. In contrast, there are also studies highlighting that some participants choose to not disclose their suicide history but are at risk (e.g., Podlogar et al., 2016) or underreport previous self-harming behavior (e.g., Khazem et al., 2021). Thus, correct disclosure of sensible personal information in self-reports also impacts the validity of the outcome variable. In addition, if suicidal behaviors are assessed with a single item, the item wording can substantially impact overall endorsement rates. Ammerman et al. (2021) found that item wording impacted endorsement consistency across a range of questions on suicide ideation, planning, and attempts as well as across different time frames. However, asking about lifetime suicide attempts such in our study was least affected by item wording.

### Potential Future Research Directions

With respect to the aforementioned limitations, it would clearly be desirable to include additional information such as a detailed medical record or the history of previous suicide attempts of an individual into the model. Such information was not available in the present data set but might exist for samples at risk of attempting suicide (e.g., patients with post-traumatic stress disorder, Bryan, 2016, or military personnel, Rozek et al., 2020). In addition, regarding the prediction of the exact time of suicidal behavior, the 3 years of the present study are too wide an interval to achieve the ultimate goal of preventing deaths by suicide on an individual level. However, we argue that suicide prevention in children or adolescents should be understood as a multi-stage process. At a first stage, screening tools for broad community samples are helpful in providing a rough estimate of the overall risk on an interindividual level. The present study hopefully adds to the knowledge about influential predictors in such an assessment. However, a screening tool cannot be used for individual diagnoses at a specific moment in time. Predicting a narrow time frame with an elevated risk of suicidal behavior for an individual requires a different assessment relying on intraindividual data including fluctuating emotional states, interpersonal problems, triggering situations, or access to common means of suicide. The main reason for this is that while there are some relatively robust factors enhancing the risk at general, acute risk of death by suicide is defined by high levels of heterogeneity in individual circumstances, calling for a more personalized modeling of states across shorter time intervals (Kaurin et al., 2022).

Thus, after selecting at-risk participants in community screenings, a closer and more tailored monitoring could be initiated at a second stage. For clinical samples, adolescents have shown high adherence to ecological momentary assessments perceiving them as positive and helpful rather than burdensome (e.g., Glenn et al., 2022). Also in these cases, prediction models that integrate dynamic risk factors of suicidal thoughts in recently discharged suicidal adolescence have evidenced promising results. For example, Czyz et al. (2021) used different combinations of the mean and variance of six risk factors (e.g., hopelessness, connectedness, and psychological pain) assessed via daily diaries for the detection of suicidal crisis. At a 1-month follow-up visit after the discharge of adolescent psychiatric inpatients, they achieved high classification performance (AUC = .91). The extent to which those results can be transferred to adolescents who have not been hospitalized after a recent suicide attempt but have been flagged by broader risk screenings, however, remains an open question that could be addressed in future research.

## Conclusion

The present analyses of longitudinal panel data showed that it is possible to predict lifetime suicide attempts in a community sample of adolescents. The results of such a screening could help to find relevant factors that can be used in an initial step of a two-stage suicide risk assessment to initiate a more fine-grained evaluation or to provide information on how to seek help. Besides previous self-harm, indicators of poor mental health and negative emotions were most indicative for lifetime suicide attempts. Because using more complex ML algorithm did not lead to improvements in predictive performance, reliably assessing relevant longitudinal information seems more promising for the improvement of suicide prediction than using even more complex statistical models. Furthermore, our results indicated shifts of variable importances across different stages of adolescence, suggesting that the assessment should be tailored to the developmental phase.

## Notes

1. Because the information on lifetime suicide attempts and the predictor variables were assessed at the same time point (for the models using data from the 17-year-olds), the modeling identifies the best indicators of past attempts, rather than assessing future behavior.
